# Histone Acetylation Dynamics Regulate Fungal Development, Pathogenicity, and Mycotoxin Biosynthesis

**DOI:** 10.3390/jof12090680

**Published:** 2026-09-10

**Authors:** Chen Gong, Zhengling Li, Daiying Xu, Daiyuan Sun, Zhaoyang Wang, Xijun Du, Yu Zhang, Xueli Qi

**Affiliations:** 1 Crop Germplasm Resources Research Institute, Henan Academy of Agricultural Sciences, Zhengzhou 450002, China; gongchen_edu@163.com (C.G.); lxlandlzl@163.com (Z.L.); duxijun2015@163.com (X.D.); 2 National Engineering Laboratory of Wheat, Key Laboratory of Wheat Biology and Genetic Breeding in Central Huanghuai Area, Ministry of Agriculture, Henan Key Laboratory of Wheat Germplasm Resources Innovation and Improvement, Zhengzhou 450002, China; 3 The Shennong Laboratory, Zhengzhou 450002, China; 4 State Key Laboratory of Crop Stress Biology for Arid Areas, College of Plant Protection, Northwest A&F University, Yangling 712100, China; xudaiying@nwafu.edu.cn (D.X.); sdybhmx1117@163.com (D.S.); 5 College of Agronomy, Henan Agricultural University, Zhengzhou 450046, China; wzy89265@163.com

**Keywords:** acetylation homeostasis, deoxynivalenol biosynthesis, chromatin remodeling, signal transduction, non-histone acetylation, epigenetic regulation

## Abstract

Histone acetylation, catalyzed by histone acetyltransferases (HATs) and reversed by histone deacetylases (HDACs), is a central epigenetic mechanism that regulates gene expression in eukaryotes. Identifying and characterizing these enzymes has rapidly advanced our understanding of this dynamic modification. In phytopathogenic fungi, HATs and HDACs modulate several cellular processes, including development, pathogenicity, and secondary metabolism. The filamentous fungus *Fusarium graminearum* causes Fusarium head blight, a destructive disease that reduces wheat yield and contaminates grain with the trichothecene mycotoxin deoxynivalenol (DON). Consequently, elucidating the regulatory networks that control fungal development, host colonization, and mycotoxin biosynthesis is essential for effective disease management. This review collates current knowledge on acetylation homeostasis in phytopathogenic fungi, with particular emphasis on *F. graminearum*, and evaluates evidence supporting a model wherein acetylation acts as a conditional regulatory hub rather than a universal transcriptional switch. Drawing on gene-deletion mutant analyses, large-scale acetylome profiling, and comparative genomics across multiple fungal species, we first cataloged the major HAT families—GNAT, MYST, CBP/p300, and TAF1—and HDAC families—Rpd3, Hos2, Hda1, and Sir2—and summarized the lysine acetylation and deacetylation sites they target, including H2BK11, H2BK16, H3K9, H3K14, H3K18, H3K23, H3K27, and H3K56. We then examine how acetylation homeostasis, defined as the dynamic balance between acetylation and deacetylation, regulates fungal morphogenesis, pathogenicity, and DON biosynthesis in *F. graminearum*, highlighting the functional interplay between HAT and HDAC complexes—exemplified by Gcn5 and Hos2 (Hdf1)—and their integration with cAMP–PKA and MAPK signaling cascades. We further analyzed how acetylation-dependent regulation intersected with proteasome-mediated protein stability, non-histone acetylation, and crosstalk with histone ubiquitination and methylation and assessed how acetylation responds to host-derived metabolites and microbial competitors, thereby extending its regulatory influence beyond canonical histone marks. Finally, comparative analyses across *Magnaporthe oryzae*, *Aspergillus* species, *Botrytis cinerea*, and *Ustilago maydis* reveal both conserved and species-specific roles of acetylation in pathogenicity. We conclude by advancing a refined conceptual model wherein acetylation operates not as a universal transcriptional on/off switch but as a conditional regulatory hub and is site-, locus-, and stage-specific and signal-responsive. Future studies that combine ChIP-seq, CUT&Tag, and multi-omics approaches will further clarify the direct molecular targets of HATs and HDACs and define their stage-specific activities during host colonization, thereby enabling the design of precise, pathogen-specific interventions. This framework offers new perspectives for developing targeted strategies to manage fungal diseases and reduce mycotoxin contamination.

## 1. Introduction

Wheat is cultivated on more than 200 million hectares worldwide across a wide range of latitudes and altitudes, under different crop rotations and production practices; these extensive cultivation areas provide favorable environmental conditions for more than 50 important diseases and pests [1], among which fungal diseases caused by biotrophic or necrotrophic fungi are predominant. Notable examples include Fusarium head blight (FHB), wheat stripe rust, wheat powdery mildew, and Fusarium crown rot, all of which threaten crop production and food safety [2]. Among the causal agents of these diseases, *Fusarium graminearum* is a filamentous fungus that infects wheat, barley, oats, corn, and other cereal crops and is the primary causal agent of FHB, which reduces both grain yield and quality while contaminating harvested grain with trichothecene mycotoxins, particularly deoxynivalenol (DON). In addition to posing food and feed safety risks, DON also facilitates fungal spread within infected plant tissues [3,4]. Thus, elucidating the mechanisms by which *F. graminearum* regulates its development, host colonization, and DON biosynthesis remains a central objective in plant pathology and fungal molecular biology research.

Among phytopathogenic fungi, *F. graminearum* has emerged as a premier model for studying the epigenetic regulation of pathogenicity, owing to three key features: (i) its genetic tractability, including well-established protocols for targeted gene deletion and complementation that enable rigorous functional validation; (ii) its well-characterized infection cycle, which encompasses distinct developmental transitions—from conidiation and spore germination to penetration, invasive hyphal growth, and secondary metabolism—that provide multiple checkpoints at which acetylation dynamics can be interrogated; and (iii) its hemibiotrophic behavior, in which an initial biotrophic phase is followed by a necrotrophic phase of tissue maceration, offering a unique system to examine how acetylation homeostasis coordinates the transition between these contrasting nutritional strategies. The above attributes make *F. graminearum* a particularly well-suited target of study for elucidating acetylation-dependent regulatory networks that are conserved across phytopathogenic fungi.

*F. graminearum* infection in wheat is a highly dynamic process [5]. Pathogenic cells continuously perceive and respond to environmental and host-derived cues, including signals from the plant surface, nutrient availability, oxidative stress, host defense metabolites, microbial competitors, and tissue-specific barriers. In response to these cues, *F. graminearum* activates transcriptional programs that regulate key processes throughout the infection cycle, including conidiation, spore germination, infection structure formation, invasive growth, secondary metabolism, and stress resistance [6,7]. For example, the G protein-coupled receptor-related genes *GIV1*–*GIV5* are significantly upregulated during plant infection and play important roles in fungal virulence [8]. The cyclic adenosine monophosphate–protein kinase A (cAMP–PKA) and mitogen-activated protein kinase (MAPK) pathways are the major components of transcriptional regulation [9,10]. However, transcription factors operate within the context of chromatin, and their access to target genes is strongly influenced by nucleosome organization and post-translational modifications (PTMs) [11].

Histone PTMs, such as methylation, acetylation, phosphorylation, and ubiquitination, play key roles in regulating various biological processes [12]; among these, histone acetylation, one of the first identified and still extensively studied in eukaryotes, involves adding acetyl groups to lysine residues, which neutralizes their positive charge, weakens histone–DNA interactions, and creates binding sites for chromatin readers. Consequently, histone acetylation is strongly linked to euchromatin formation and transcriptional activation; however, viewing acetylation as a simple on/off switch for gene expression does not explain the complex phenotypes observed in filamentous fungal pathogens. If acetylation only activated transcription, disrupting histone acetyltransferases would be expected to cause defects, while disrupting histone deacetylases would not, or would have opposite effects. In practice, genetic disruption of either histone acetyltransferases or histone deacetylases often leads to pleiotropic defects in growth, development, and virulence, as seen with *FgGCN5* and *FgHDF1* [13,14]; furthermore, individual histone deacetylases can repress certain genomic loci while also being necessary for the full expression of others, especially within secondary metabolite gene clusters [15,16,17]. The above findings suggest that the effects of acetylation depend not simply on the presence of acetyl marks, but also on the dynamic balance between acetylation and deacetylation—a concept we refer to as acetylation homeostasis, an understanding of which is crucial for unraveling the mechanisms underlying fungal pathogenicity.

To systematically examine the role of acetylation homeostasis in phytopathogenic fungi, this review draws on a curated selection of the core literature, identified from searches of the Web of Science and PubMed databases, with emphasis on studies published over the past two decades while also incorporating seminal foundational studies. The search terms included combinations of “*Fusarium graminearum*,” “histone acetylation,” “histone acetyltransferase,” “histone deacetylase,” “pathogenicity,” “deoxynivalenol,” “chromatin remodeling,” and “phytopathogenic fungi,” with additional references identified by manually screening key reviews and original research articles. Rather than attempting an exhaustive compilation, we prioritized studies that directly addressed the roles of HATs or HDACs in fungal development, pathogenicity, or secondary metabolism, with emphasis on *F. graminearum* and comparative analyses across other phytopathogenic species. Drawing on gene-deletion mutant analyses, large-scale acetylome profiling, and comparative genomics, we aim to synthesize current knowledge on acetylation homeostasis in phytopathogenic fungi and to evaluate the evidence supporting a model wherein acetylation functions as a conditional regulatory hub, rather than a universal transcriptional switch. We first introduce the major HAT and HDAC systems and summarize the lysine acetylation and deacetylation sites they target in fungal chromatin. We then discuss *F. graminearum* as a key model for elucidating how acetylation and deacetylation regulate fungal development, pathogenicity, and DON biosynthesis. Finally, we compare current knowledge across other phytopathogenic fungi and highlight emerging research directions for elucidating acetylation-dependent regulatory networks and their contributions to fungal pathogenicity.

Throughout this review, we explicitly distinguish between regulatory mechanisms that have been directly demonstrated in *F. graminearum* via targeted mutant analyses and those that have been inferred from comparative genomics or studies in other fungal species, a distinction intended to clarify the evidence base supporting the acetylation homeostasis model and to identify areas where experimental validation in *F. graminearum* or other phytopathogens is still needed. Where findings from other fungi are used to infer mechanisms in *F. graminearum*, we indicate the source organism and the degree of functional conservation supporting the inference.

## 2. Histone Acetylation, Deacetylation, and Acetylation Homeostasis

Histone acetylation is a dynamic epigenetic modification, controlled by the antagonistic activities of HATs and HDACs, and plays an important role in regulating gene expression in filamentous fungi. The catalytic domains of the major HAT families—GCN5-related N-acetyltransferase (GNAT), MOZ-YBF2/SAS3-SAS2-TIP60 (MYST), CREB-binding protein (CBP/p300), and TBP-associated factor 1 (TAF1)—are evolutionarily conserved [18], yet their biological functions are remarkably diverse, enabling coordination of a broad spectrum of developmental and pathogenic processes. Rather than acting as isolated enzymes, HATs typically function as catalytic subunits within large multiprotein complexes, whose architectural components determine substrate specificity and genomic recruitment; this principle is exemplified by the GNAT family member GCN5, which serves as the catalytic core of the SAGA, ADA, and SLIK/SALSA complexes and acetylates H2BK11, H2BK16, and H3K9, H3K14, H3K18, H3K23, and H3K27 [19,20] (Table S1). In *F. graminearum*, *FgGCN5* is essential for vegetative growth, conidiation, and virulence, as demonstrated directly in functional assays [13]. Similarly, based on studies in other fungi, the *Magnaporthe oryzae GCN5* ortholog regulates appressorium-mediated host penetration [21,22]. The MYST family acetyltransferase Sas3, as a component of the NuA3 complex, specifically mediates H3K9 and H3K14 acetylation to maintain genome integrity and regulate secondary metabolism in *Saccharomyces cerevisiae* [23], whereas ESA1, within the NuA4 complex, targets H4 and H2A.Z to control cell-cycle progression [24,25,26]. Furthermore, as proposed based on comparative genomics, the TAF1-family acetyltransferase Tip60 participates in the DNA damage response, and the GNAT member Rtt109, activated by chaperones Asf1 and Vps75, predominantly acetylates H3K56 to ensure genomic stability during rapid growth in *Candida albicans* [27,28,29,30]. Collectively, these examples demonstrate that HAT diversity is organized not around catalytic specificity alone, but around complex-associated recruitment mechanisms that link distinct acetylation marks to specific biological contexts.

Filamentous fungi possess two major classes of HDACs: Zn^2+^-dependent classical enzymes and NAD^+^-dependent sirtuins, which function as critical epigenetic rheostats. Classical HDACs are grouped into conserved families—Rpd3, Hos2 (Class I), Hda1 (Class II), and Sir2 (Class III)—and, similar to HATs, operate within multi-subunit complexes, rather than as solitary catalysts [31,32,33,34]. HOS2 forms the catalytic core of the Set3C complex (comprising SET3, SNT1, HOS2, CPR1, HST1, and HOS4) and deacetylates nucleosomes near meiotic genes, whereas RPD3, functioning within the Rpd3L complex built around scaffold proteins, targets promoters of hypha-specific genes whose hyperacetylation drives the yeast-to-hypha morphological transition in *C. albicans* [35,36,37,38,39]. Although HDACs are classically associated with transcriptional repression, their functions extend far beyond silencing: in *Aspergillus* species, HDACs suppress ectopic transcription within secondary metabolite gene clusters (e.g., sterigmatocystin), ensuring that toxin biosynthesis remains tightly coupled to environmental cues [38], and certain HDACs can also promote the expression of specific gene sets involved in fungal survival and environmental interaction [40]; this functional duality—repressing aberrant transcription at some loci, while permitting or facilitating expression at others—explains why HDACs should be viewed not as simple silencers, but as finely tuned regulators of chromatin turnover.

Based on these findings, we propose the concept of acetylation homeostasis, which challenges the assumption that increased histone acetylation is invariably beneficial for transcription or pathogenicity. Fungi do not necessarily benefit from globally elevated acetylation; rather, they require precisely tuned acetylation states at specific lysine residues and defined genomic loci. Under this framework, loss of HATs not only reduces acetylation at direct target sites, but may indirectly alter other chromatin modifications and disrupt recruitment of chromatin-associated complexes. Similarly, HDAC loss or overexpression can derepress a subset of genes while simultaneously impairing others. Collectively, these observations indicate that HATs and HDACs function as integral components of a coordinated regulatory network, rather than as simple, mutually antagonistic on/off switches.

A critical synthesis of the current literature reveals both consistencies and contradictions across fungal species. For example, consistent results are observed regarding the antagonism between Gcn5 and Rpd3, which is demonstrated directly in *F. graminearum* [13,41] and supported by studies on *S. cerevisiae* and *C. albicans* [35,36,37,38,39]. However, contradictory findings exist regarding specific histone marks; for instance, the function of H3K9ac varies significantly among species, and Hos2 homologs show divergent phenotypes, ranging from essential virulence factors in *F. graminearum* to dispensable regulators in other models [14,35]. Furthermore, there is insufficient evidence to fully map the non-histone acetylome in many plant pathogens, leaving gaps in our understanding of how acetylation homeostasis integrates with post-translational signaling networks.

## 3. Pathogenicity of *F. graminearum* Is Regulated by Histone Acetylation Homeostasis

*F. graminearum* serves as an appropriate model system for elucidating the mechanisms of histone acetylation homeostasis, due to its high genetic tractability, well-characterized infection cycle, and hemibiotrophic behavior, which provides multiple contexts in which chromatin regulators can exert distinct and coordinated effects. As such, F. *graminearum* provides a useful model for investigating how histone acetylation homeostasis regulates fungal pathogenicity.

Recent research has revealed that histone acetylation serves as a central epigenetic mechanism that coordinates diverse biological processes, operating not as a uniform switch, but through a sophisticated and modular regulatory network [42]. Large-scale lysine acetylome analyses have further highlighted this complexity by identifying hundreds of acetylated sites across diverse substrates [43]. Notably, studies in *F. graminearum* have identified seven non-histone proteins associated with fungal virulence or deoxynivalenol (DON) biosynthesis, including the transcription factors AreA, Rlm1, and StuA, and the protein kinases Fus3, Gpmk1, Sch9, and Mgv1 [44,45,46,47,48]. HATs themselves also exhibit functional specialization at the chromatin level; for example, deletion of *FgGCN5* completely abolishes DON biosynthesis and conidiation [13], whereas deletion of *FgEAF7*, a subunit of the NuA4 complex, severely impairs vegetative growth without substantially affecting pathogenicity [46]. Furthermore, the acetylases FgGcn5 and FgSas3 display distinct substrate preferences: FgGcn5 primarily acetylates H3K9, H3K18, and H3K27, whereas FgSas3 preferentially promotes H3K4 acetylation. H3K14 acetylation can be regulated cooperatively by both enzymes [41]. In other *Fusarium* species, mutations affecting the N-terminal residues of H3K18 have been shown to reduce toxin accumulation [49]. Moreover, the acetylation-dependent network is itself subject to post-translational control in *F. graminearum*. The E3 ubiquitin ligase Tom1 promotes Gcn5 degradation, whereas the de-ubiquitinating enzyme Ubp14 stabilizes Gcn5 [50]. Therefore, FHB pathogenicity is determined not only by histone acetylation, but also by the proteostasis of the modifying enzymes themselves.

**Figure 1:**
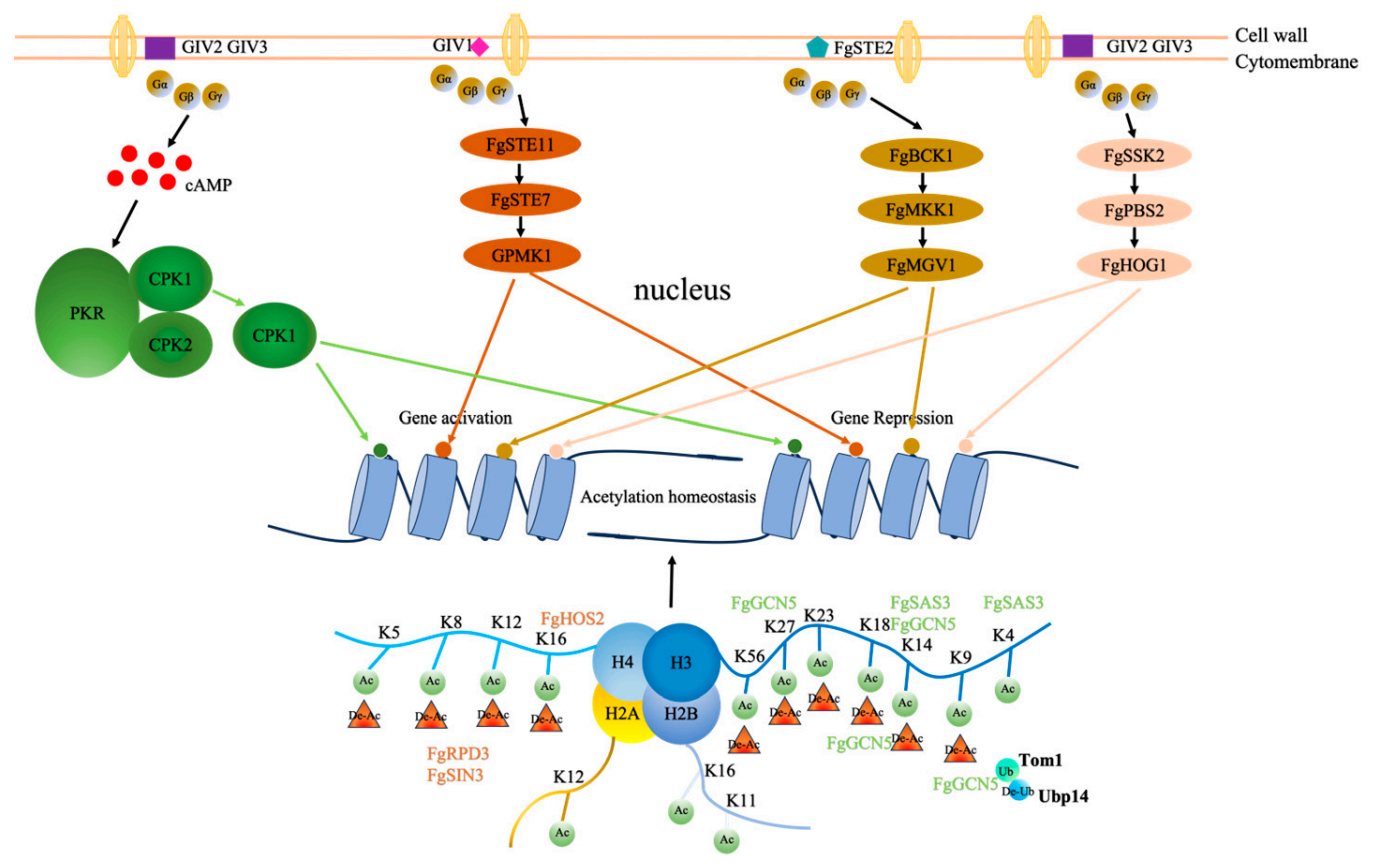
Histone acetylation homeostasis in *F. graminearum*. Note: DON is a key virulence determinant in fungal infections. Within the regulatory network that governs DON biosynthesis, histone acetylation occupies a central position in a precisely calibrated dynamic equilibrium; this balance is maintained via coordinated chromatin modification processes, rather than a simple binary mechanism. Functional crosstalk between HAT and HDAC activities establishes a basal chromatin landscape that, in turn, directly or indirectly modulates *TRI* gene expression. External signals are perceived by the membrane proteins GIV1–GIV5 and FgSTE2, and transmitted via G-proteins. Thereafter, they are subsequently propagated via the cAMP–PKA and MAPK signaling cascades, which collectively regulate histone acetylation and deacetylation dynamics. In this figure, green spheres denote histone acetylation, orange triangles indicate histone deacetylation, bright green spheres represent ubiquitination, and bright blue spheres represent de-ubiquitination.

Although HATs play important roles in fungal development and pathogenicity, HDACs are likewise critical for maintaining acetylation homeostasis and, thereby, regulating fungal pathogenicity (Figure 1). The functional importance of deacetylation extends beyond its canonical role in transcriptional repression. For example, as demonstrated directly in *F. graminearum*, the HOS2 ortholog HDF1 is required for conidiation, sexual reproduction, and DON biosynthesis [14]; this HAT–HDAC functional interplay is particularly evident in the antagonistic relationship between the Fng1–NuA4 HAT complex and the Rpd3 HDAC system. Although the deletion of *FNG1* causes severe developmental defects that can be partially rescued by suppressing Rpd3 components (*FgRPD3* and *FgSIN3*) [51], this incomplete restoration indicates that simply reducing HDAC activity is insufficient to fully reconstitute the precise chromatin landscapes required for fungal infection. This regulatory complexity is further illustrated by scaffold proteins such as FNG3, which interact with both the NuA3 HAT and Rpd3 HDAC complexes to fine-tune local acetylation states [52]. Beyond maintaining enzymatic equilibrium, HDAC complexes can function as regulatory hubs that link signal transduction to chromatin remodeling. As demonstrated directly in *F. graminearum*, the Hos2/Set3 complex subunit FgSNT1 mediates crosstalk with the cAMP–PKA pathway via phosphorylation by Cpk1, the catalytic subunit of PKA [53]. In S. cerevisiae, the Hos2p complex is required for the activation of the Mpk1p/Slt2p cell-integrity kinase cascade in response to secretory stress [54]; however, whether the Mpk1p/Slt2p homolog FgMGV1 similarly functions in concert with HOS2 in *F. graminearum* remains hypothetical. Nevertheless, the finding that the PKA catalytic subunit Cpk1 phosphorylates its direct downstream target FgSFL1, which, in turn, regulates virulence [53], further illustrates the highly integrated network in which signaling cascades, protein stability, and chromatin dynamics collectively govern fungal pathogenicity.

**Figure 2:**
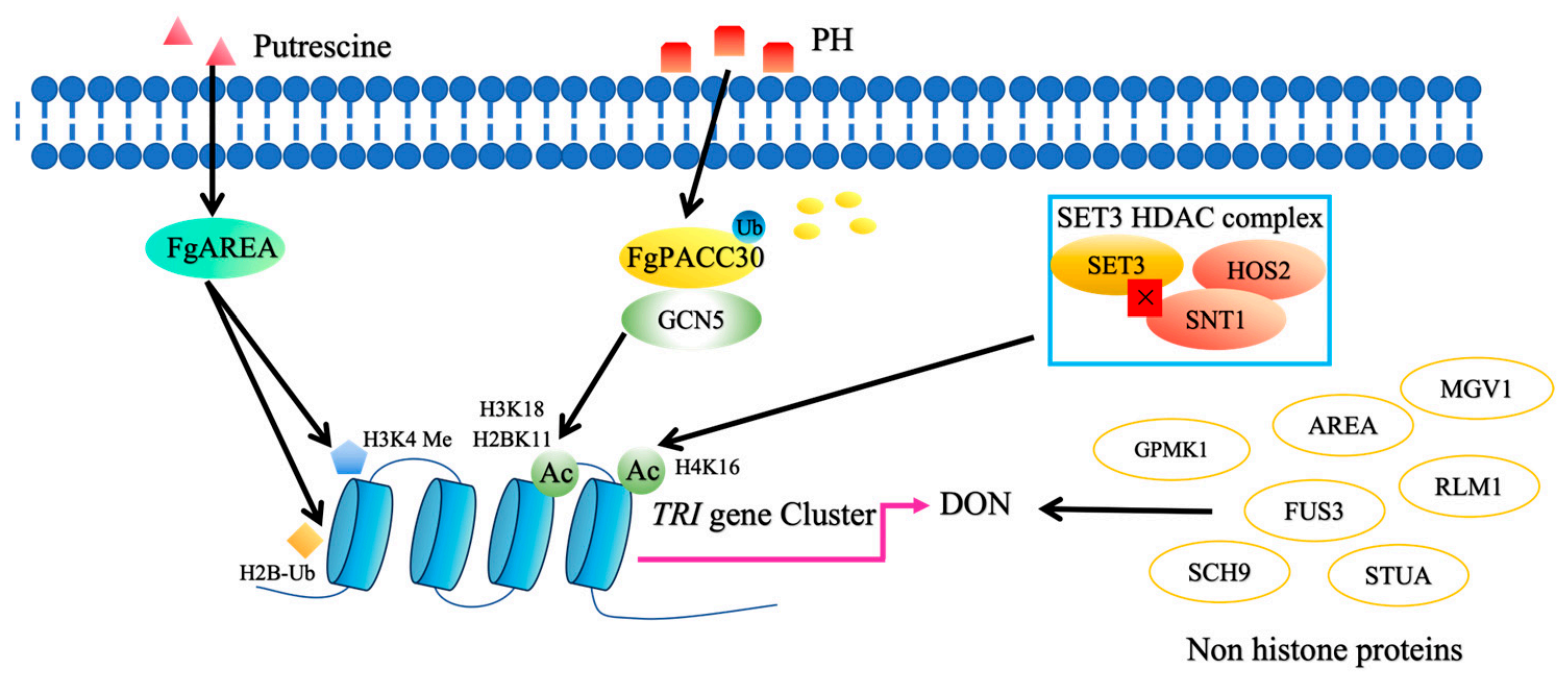
DON biosynthesis regulated by multiple chromatin-modifying activities in *F.graminearum*. Note: This diagram depicts the regulatory network linking histone acetylation, additional epigenetic modifications, and signaling pathways in the control of DON biosynthesis. The *TRI* gene cluster is subject to multiple layers of regulation. First, changes in external pH reduce the degradation of FgPACC30 following ubiquitination, thereby stabilizing its association with GCN5 and modulating histone acetylation. Second, putrescine induces the expression of *FgAREA*, which directly governs the histone-mediated regulation of DON via H3K4 methylation and H2B ubiquitination. Third, truncation of the core component of the SET3 complex, involving loss of the last 98 amino acids at its C-terminus, alters its interaction profile and promotes binding to HOS2, consequently affecting histone H4K16 activity and regulating DON synthesis. In addition to these histone-based mechanisms, non-histone factors also exert direct control over DON production. Green spheres represent histone acetylation; yellow squares represent histone H2B monoubiquitination; blue spheres represent ubiquitination; purple diamonds represent methylation; pink triangles represent putrescine; and red squares represent pH.

DON is a major virulence-associated factor in fungal infections. Within the regulatory network controlling DON biosynthesis, histone acetylation does not function as a simple binary on/off switch, but instead functions as a pivotal component of a dynamic equilibrium governed by multiple chromatin-modifying activities. The reciprocal activities of HATs and HDACs therefore contribute to establishing and maintaining a basal permissive chromatin state that directly or indirectly regulates the expression of *TRI* genes (Figure 2). Deletion of *HOS2* results in a significant reduction in virulence and DON production. Moreover, *FgSNT1*, a core component of the Hos2/Set3 HDAC complex, undergoes structural changes following truncation of its C-terminal 98 amino acids, which alters the interaction model of the complex, shifting its association from SET3 toward HOS2 and, consequently, affecting both infection capacity and DON accumulation [14,53]. Increasing evidence further indicates that histone acetylation and deacetylation are tightly constrained by their crosstalk with other epigenetic modifications and environmental signaling pathways. Regulation of pH sensing provides a clear example of this integrated control. Recent studies have shown that the transcription factor FgPACC30 acts as a negative regulator of DON biosynthesis by directly binding to the promoter of *FgTRI1*. Rather than simply blocking the access of transcriptional activators through steric obstruction, FgPACC30 represses the local histone acetylation machinery, thereby establishing a repressive chromatin configuration at the *FgTRI1* locus; this finding demonstrates that the acetylation state established by enzymes such as Gcn5 can be modulated by environmental pH signals, suppressing toxin production under unfavorable conditions [55]. The chromatin landscape is likewise responsive to host-derived metabolites. For example, putrescine stimulates DON production by initiating chromatin remodeling events. In the presence of putrescine, the nitrogen-responsive transcription factor FgAREA is recruited to the *TRI* locus, where it promotes acetylation of H2B monoubiquitination (H2Bub1) and H3K4 di- and trimethylation (H3K4me2/3), thus reaching an actively transcribed chromatin state [56]. Similarly, studies in *Fusarium pseudograminearum* have shown that histone acetylation at the *TRI* loci acts together with specific histone methylation marks to maintain robust transcriptional activity. The functional importance of this fine balance becomes even more evident when considering residue-specific histone modifications. Studies in related *Fusarium* species have shown that targeted mutations of individual N-terminal H3 lysine residues, including K9, K14, K18, and K23, produce distinct phenotypic effects, ranging from altered growth rates to severe pathogenic defects. The above observations imply that the *TRI* gene cluster is regulated by a precise combinatorial histone code, rather than by a single modification [48]. Taken together, these findings demonstrate that understanding the biosynthesis of mycotoxins requires a more complete understanding beyond a single histone acetylation or deacetylation perspective. Instead, it requires a framework that considers the coordinated dynamics of multiple histone modifications and chromatin accessibility in response to environmental events.

## 4. Multiple Roles of Acetylation in Fungal Pathogenicity

In Section 3, we introduced the nitrogen regulator FgAREA, which recruits histone-modifying enzymes to the *TRI* gene cluster in response to putrescine [56], a finding that provides a striking example of how fungal pathogens can exploit host-derived metabolites. During infection, the plant defense metabolite putrescine is not simply encountered or tolerated by *F. graminearum*, but can be repurposed as a signal to activate fungal virulence. Through this mechanism, a host-derived chemical cue is effectively converted into an epigenetic switch that promotes fungal infection. Conversely, microbial competitors can use epigenetic antagonists as chemical agents; for example, as demonstrated directly in *F. graminearum*, the biocontrol bacterium Pseudomonas piscium produces phenazine-1-carboxamide (PCN), which directly targets and impairs the activity of the core HAT FgGCN5, thereby disrupting the chromatin remodeling required for fungal growth [57,58].

In addition to its role in altering chromatin structure in response to environmental factors, histone acetylation also functions as a rapid post-translational switch for non-histone proteins, an additional layer of regulation that enables fungi to bypass the relatively slow transcriptional process and directly control virulence factors at the protein level. Acetylome analyses of *F. graminearum* have identified numerous acetylated non-histone substrates, including key MAPKs and transcription factors, which, in turn, may directly alter the activity or stability of pathogenic proteins, thereby adding a post-translational layer of regulation to the transcriptional programs controlled by GCN5 [41,50,59,60,61,62]. While the acetylation of specific non-histone targets is demonstrated directly in *F. graminearum*, the broader functional consequences of many non-histone acetylation events remain inferred from proteomic datasets and require further validation.

Taken together, these findings indicate that the functions of histone acetylation in filamentous fungi include the regulation of gene expression through acetylation and deacetylation, as well as the utilization of host secondary metabolites and direct control of non-histone proteins through acetylation. These functions of histone acetylation are conserved across phytopathogenic fungi. For example, as demonstrated directly in *M. oryzae*, the acetyltransferase MoHat1 directly acetylates the autophagy proteins MoATG3 and MoATG9 to control appressorium development and infection [63]. Thus, this evidence shows that the acetylation network extends its regulatory influence far beyond canonical histone marks, enabling fungal pathogens to respond rapidly to host invasion through coordinated transcriptional and post-translational mechanisms.

## 5. Comparative Pathogenicity: Acetylation in Other Phytopathogenic Fungi

While our discussion of *F. graminearum* illustrates how histone acetylation homeostasis integrates chromatin regulation, environmental sensing, and pathogenicity, it is essential to distinguish mechanisms demonstrated directly in *F. graminearum* from those inferred from other fungal models. The fungal kingdom exhibits profound diversity in how acetylation homeostasis is achieved. In *F. graminearum*, direct genetic and biochemical evidence has established that the acetyltransferase FgGcn5 and the deacetylase FgHos2 are non-redundant regulators of deoxynivalenol (DON) biosynthesis and virulence. However, in many other phytopathogens, the specific molecular wiring of these pathways remains inferred based on conserved domain architecture, rather than direct functional validation.

A critical synthesis of the current literature reveals both conserved principles and notable exceptions across species. Consistently, Class I and II HDACs act as central modulators of secondary metabolism and virulence. For example, in *Fusarium fujikuroi*, FfHDA1 and FfHDA2 regulate virulence and secondary metabolism [64], while in Botrytis cinerea, BcRPD3 is critical for infection structure formation and oxidative stress responses [65]. Similarly, the cAMP–PKA signaling pathway frequently intersects with acetylation machinery, as seen in *Ustilago maydis*, where HOS2 regulates mating-type genes downstream of cAMP–PKA signaling [66]. However, contradictory results highlight significant functional divergence. In *Aspergillus nidulans*, the ortholog of GCN5 is dispensable for virulence despite its critical role in conidiation and stress responses [67], contrasting sharply with the essential virulence role of FgGCN5. Furthermore, MYST-family proteins in Aspergillus flavus demonstrate opposing functions: MYSTA (SAS2 ortholog) negatively regulates aflatoxin B1 production via H4K16 acetylation, whereas MYSTB (SAS3 ortholog) positively regulates it via H3K14, H3K18, and H3K23 acetylation [68]. These exceptions reinforce the suggestion that HDACs and HATs cannot be universally categorized as simple transcriptional repressors or activators; rather, they function as dynamic, context-dependent modulators of chromatin architecture.

In *Botrytis cinerea*, BcRPD3-mediated deacetylation is critical for vegetative growth, infection structure formation, oxidative stress responses, and virulence [65]. In the smut fungus *U. maydis*, HOS2 regulates virulence by controlling mating-type genes and functions downstream of cAMP–PKA signaling [66]. Sirtuin-related proteins in *U. maydis* also regulate the expression of pathogenicity-associated genes, although their mechanisms of action may differ from those of classical histone deacetylation; recent studies of other pathogens further demonstrate this diversity. For instance, in *Verticillium dahliae*, histone acetylation dynamics are critical for the formation of microsclerotia, long-term survival structures required for soil persistence and host invasion [69]. Similarly, in *F. oxysporum*, specific HATs are required for the appropriate expression of effector genes that facilitate colonization of host xylem vessels [70,71]. In *M. oryzae*, SIR2 contributes to the suppression of rice defense responses, whereas MoHAT1 regulates appressorium function through the acetylation of autophagy-related proteins [63,72]. Collectively, these findings suggest that, while the core acetylation machinery is evolutionarily conserved, its specific targets and biological consequences have diversified to meet the distinct ecological requirements of individual pathogens, ranging from necrotrophy to biotrophy, a diversity that underscores the necessity of validating the acetylation homeostasis framework within each pathosystem, rather than extrapolating mechanisms uncritically from model organisms.

## 6. Diverse Acylations and Species-Specific Regulatory Roles

Beyond canonical histone acetylation, emerging evidence indicates that a broader repertoire of acylation mechanisms contributes to fungal development and pathogenicity. Direct experimental evidence from *Alternaria alternata* demonstrates that multiple HATs and HDACs are required for virulence, conidiation, stress tolerance, and DNA damage repair [73]. Similarly, direct evidence in *A. flavus* shows that Rtt109-mediated H3K9 acetylation affects morphogenesis, aflatoxin biosynthesis, and pathogenicity [74]. Proposed interpretations, based on structural homology and preliminary biochemical assays, suggest that non-acetyl acylations also play significant roles. For instance, AfNGG1-mediated histone 2-hydroxyisobutyrylation of H4K5 and H4K8 has been linked to fungal development and aflatoxin production [75], and the epigenetic reader SNTB in *A. flavus* is proposed to regulate development and toxin biosynthesis through antioxidant response mechanisms [76].

In *Colletotrichum gloeosporioides*, CgHAT1 has been shown to regulate H4K5 and H4K12 acetylation, contributing to growth, conidiation, appressorium formation, and pathogenicity [77]. Collectively, these findings demonstrate that acetylation and related acylation mechanisms are widely involved in pathogenic development and toxin biosynthesis. However, a critical limitation of current knowledge is the lack of comprehensive, site-specific acylome profiling across diverse fungal species. Differences in fungal behaviors, host niches, infection structures, metabolite repertoires, and gene-cluster organization result in substantial variation in the downstream gene-expression programs regulated by these modifications. Consequently, while the prevalence of diverse acylations is established, the precise functional boundaries between canonical acetylation and emerging acylation marks remain poorly defined. Addressing these knowledge gaps requires moving beyond correlative observations to establish causal, site-specific regulatory networks, which will be essential for understanding how acetylation homeostasis integrates with broader epigenetic landscapes (Section 7).

## 7. A New Conceptual Model of Fungal Acetylation

Accumulating evidence from *Fusarium* species and other phytopathogenic fungi supports a revised and more nuanced model of acetylation, which we term acetylation homeostasis; this model rests on five key dimensions. First, acetylation is site-specific: histone marks such as H3K9ac, H3K14ac, and H3K27ac do not have identical functions, and different HATs contribute to distinct acetylation patterns at defined lysine residues. Second, acetylation is locus-specific: secondary metabolite clusters, stress-response genes, and infection-associated genes can respond differently to changes in local acetylation status, as demonstrated by the dual repressive and activating roles of individual HDACs across distinct genomic regions [15,16,17,38,40]. Third, acetylation is stage-specific: the chromatin states required for vegetative growth may differ substantially from those required for conidiation, sexual reproduction, and invasive growth. Fourth, acetylation is signal-responsive and can be altered in response to host-derived metabolites, bacterial compounds, and conserved signaling pathways. Fifth, acetylation is incorporated into other PTMs, including H2B ubiquitination, H3K4 methylation, phosphorylation, and ubiquitination, forming a multilayered regulatory landscape, rather than an isolated modification system.

These five dimensions collectively challenge the simplistic assumption that increased histone acetylation invariably results in increased transcription. A more accurate model proposes that phytopathogenic fungi use acetylation homeostasis to establish dynamic and conditional chromatin states, which can permit, restrict, or fine-tune specific gene-expression programs based on the regulatory context, rather than acetylation levels alone. Under this framework, HATs and HDACs function not as simple, mutually antagonistic on/off switches, but as integral components of a coordinated regulatory network that combine developmental cues, environmental signals, and inter-modification crosstalk to maintain context-appropriate chromatin states.

## 8. Future Perspectives and Conclusions

### 8.1. Linking Methodological Approaches to Unresolved Biological Questions

To advance the acetylation homeostasis framework, future research must explicitly link methodological advances to specific unresolved biological questions. Most current studies rely on gene-deletion mutants, which reveal biological function, but not direct molecular targets; to address this, ChIP-seq and CUT&Tag must be applied to directly map the genomic occupancy of chromatin regulators and the distribution of specific marks, addressing the question: What are the direct, genome-wide targets of specific HATs and HDACs during pathogenic development? When combined with time-resolved infection experiments and systematic tissue sampling, these approaches can capture temporal dynamics, addressing the question: How do chromatin states remodel dynamically during host colonization, and which acetylation changes drive pathogenicity, versus merely accompanying developmental transitions?

### 8.2. Addressing Functional Redundancy and Non-Histone Acetylation

A critical unresolved question concerns functional redundancy among HATs and HDACs. Single-gene deletions often fail to produce discernible phenotypes due to compensatory activity. Systematic combinatorial deletion strategies and conditional expression systems are required to unmask latent functions, addressing the question: To what extent do paralogous acetylation regulators exhibit functional redundancy, and what are their collective contributions to the fungal acetylome? Furthermore, while phosphoproteomics and metabolomics have identified numerous non-histone acetylation candidates, site-directed mutagenesis and targeted biochemical assays are needed to establish causality, addressing the question: Which non-histone acetylation events are functionally consequential for pathogenicity, and how do they link with metabolic and signaling networks?

### 8.3. Multi-Omics Integration and the Acetylation Homeostasis Roadmap

Integrated chromatin profiling combined with RNA-seq, phosphoproteomics, and metabolomics provides a comprehensive view of the pathogenic regulatory network. Coupling acetylome profiling with transcriptomic and metabolomic data across multiple developmental stages enables the construction of predictive models, addressing the question: How do multiple layers of regulation converge to control fungal development and pathogenicity in the host environment?

In conclusion, histone acetylation has emerged as a central but highly context-dependent regulatory mechanism in phytopathogenic fungi. In *F. graminearum*, the interplay between HATs, HDACs, cAMP–PKA signaling, and proteasome stability contributes to the control of fungal development, virulence, and DON biosynthesis. As research progresses from global measurements toward site-specific, multilayered regulatory models, the concept of acetylation homeostasis provides a unifying framework for understanding these complex processes. This review highlights potential strategies for managing crop diseases and mitigating mycotoxin contamination by targeting specific regulatory interfaces, rather than broadly inhibiting enzymatic activities. Future research should build on these insights to identify pathogen-specific vulnerabilities and develop precise, targeted interventions against phytopathogenic fungi, ultimately translating fundamental epigenetic knowledge into sustainable agricultural solutions.

## Data Availability

No new data were created or analyzed in this study. Data sharing is not applicable to this article.

## Supplementary Materials

The following supporting information can be downloaded at: https://www.mdpi.com/article/10.3390/jof12090680/s1, Table S1, Histone modification sites and related genes covered in this review.

## Abbreviations

The following abbreviations are used in this manuscript:

cAMP	cyclic adenosine monophosphate
CBP/p300	CREB-binding protein
DON	deoxynivalenol
FHB	*Fusarium* head blight
GNAT	GCN5-related N-acetyltransferase
HAT	histone acetyltransferase
HDAC	histone deacetylase
MAPK	mitogen-activated protein kinase
MYST	MOZ-YBF2/SAS3-SAS2-TIP60
PCN	phenazine-1-carboxamide
PKA	protein kinase A
PTM	post-translational modifications
TAF	TBP-associated factor
SAS	Something About Silencing
Rpd	Reduced Potassium Dependency
Rtt109	Regulator of TY1 Transposition Gene Product 109
