# Supplementary material for: Histone Acetylation Dynamics Regulate Fungal Development, Pathogenicity, and Mycotoxin Biosynthesis
Source: J Fungi (Basel). 2026 Sep 10;12(9):680. doi: 10.3390/jof12090680 (PMC13608603; doi:10.3390/jof12090680)
Supplement: Supplementary file 1 [file jof-12-00680-s001.zip › jof-4514348-supplementary.pdf]

Table S1. Histone modification sites and related genes covered in this review.

Type	Genes	Modified Sites	Fungi	Ref.
Histone acetyltransferases	*GCN5*	H2BK11, H2BK16, H3K9, H3K14, H3K18, H3K23, H3K27	*Saccharomyces cerevisiae*	[18,19]
	SAS3	H3K9, H3K14		[22]
	ESA1	H4K16, H2A.Z		[24–26]
	Rtt109	H3K56		[27–30]
	FgGCN5	H3K9, H3K18, H3K27, H3K14	*Fusarium graminearum*	[51]
	FgSAS3	H3K4, H3K14		[41]
	MYSTA	H4K16	*Aspergillus flavus*	[68]
	MYSTB	H3K14, H3K18, H3K23		[68]
	RTT109	H3K9		[74]
	AfNGG1	H4K5, H4K8		[75]
	CgHAT1	H4K5, H4K12	*Colletotrichum gloeosporioides*	[77]
Histone deacetylases	*FgHOS2*	H4K16	*Fusarium graminearum*	[53]
Non-histone acetylation	*FgARA*	H2Bub1	*Fusarium graminearum*	[56]
